# Wavefront shaping through emulated curved space in waveguide settings

**DOI:** 10.1038/ncomms10747

**Published:** 2016-02-22

**Authors:** Chong Sheng, Rivka Bekenstein, Hui Liu, Shining Zhu, Mordechai Segev

**Affiliations:** 1 National Laboratory of Solid State Microstructures & School of Physics, Collaborative Innovation Center of Advanced Microstructures, Nanjing University, Nanjing, Jiangsu 210093, China; 2 Department of Physics and Solid State Institute, Technion, Haifa 32000, Israel

## Abstract

The past decade has witnessed remarkable progress in wavefront shaping, including shaping of beams in free space, of plasmonic wavepackets and of electronic wavefunctions. In all of these, the wavefront shaping was achieved by external means such as masks, gratings and reflection from metasurfaces. Here, we propose wavefront shaping by exploiting general relativity (GR) effects in waveguide settings. We demonstrate beam shaping within dielectric slab samples with predesigned refractive index varying so as to create curved space environment for light. We use this technique to construct very narrow non-diffracting beams and shape-invariant beams accelerating on arbitrary trajectories. Importantly, the beam transformations occur within a mere distance of 40 wavelengths, suggesting that GR can inspire any wavefront shaping in highly tight waveguide settings. In such settings, we demonstrate Einstein's Rings: a phenomenon dating back to 1936.

## Abstract (web summary)

Wavefront shaping is typically carried out outside the medium within which the beam is propagating. Sheng *et al.* exploit concepts inspired by General Relativity for wavefront shaping within optical waveguide settings, constructing narrow collimated beams and shape-preserving beams accelerating on arbitrary trajectories

## Graphical abstract

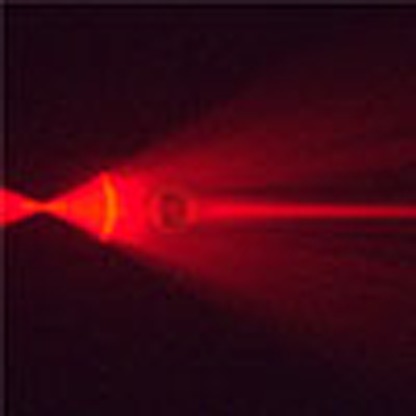

General electromagnetic (EM) beams propagating through linear homogenous media experience diffraction broadening. However, many applications would greatly benefit from having beams that remain very narrow or shape-invariant for large distances. The past two decades have witnessed remarkable progress in wavefront shaping specifically for the purpose of generating non-diffracting beams, such as shape-preserving Bessel beams^1^ and accelerating beams in free space^2^,^3^,^4^,^5^, in plasmonics^6^,^7^,^8^,^9^ and even in nonlinear materials^10^,^11^,^12^,^13^,^14^,^15^. The concept of shape-invariant wavepackets was extended beyond EM waves, for example to shaping wavefunctions of electrons^16^,^17^,^18^,^19^ and generating shape-invariant acoustic beams^20^,^21^, and even accelerating surface water gravity waves^22^. All of these shape-invariant wavepackets are not square integrable (they carry infinite power), hence physically they must be truncated, which implies that they stay non-diffracting only for a finite distance^2^. In a similar vein, there are other kind of beams which are a priori designed to stay shape-invariant only for a finite distance, for example, the cosine-Gauss beams^23^ and a class of beams that propagate on arbitrary curved trajectories^5^,^24^,^25^. Naturally, all of these beams require wavefront shaping: the launch beam must be shaped in a specific structure (amplitude and phase), to stay non-diffracting for the specified distance.

Wavefront shaping for generating non-diffracting optical beams can be achieved by various methods, ranging from annular slits^1^, axicon lenses^26^, computer generated holograms^24^, spatial light modulators^3^,^28^, gratings^7^,^23^,^29^, metasurfaces^30^,^31^,^32^ and diffraction from nanoparticles^4^,^33^. Importantly, non-diffracting beams can also be generated in inhomogeneous media such as photonic crystal slabs^34^,^35^,^36^,^37^,^38^, photonic crystals^39^,^40^ and photonic lattices^41^. All these too require wavefront shaping, that is typically done externally, outside the medium within which the beam is propagating. However, wavefront shaping can also be done by shaping the EM environment in which the wave is propagating^42^,^43^. The fact that the propagation of EM waves in static curved space is analogous to that in inhomogeneous media^42^,^43^,^44^ is the underlying principle of emulating general relativity (GR) phenomena in transformation optics^42^,^43^,^45^,^46^,^47^,^48^,^49^,^50^,^51^,^52^. In transformation optics, the permittivities and permeabilities are structured to vary according to the curvature of space^53^,^54^,^55^,^56^,^57^,^58^,^59^, giving rise to unique trajectories^55^,^56^,^57^,^60^ and controlling the diffraction of light^61^,^62^.

Here, we show that using ideas inspired by GR yields efficient beam shaping in waveguide settings. The concept is general, applicable to many cases where wavefront beam shaping in a waveguide platform is required. First, we fabricate the micro-structured optical waveguide with the specific refractive index emulating the curved space environment generated by a massive gravitational object. This dielectric structure yields a very narrow beam that remains non-diffracting for many Rayleigh lengths. Second, with the same experimental system, we demonstrate the Einstein's rings phenomenon, matching Einstein's 80 years old formula. Finally, we present a general formalism to transform Gaussian beams to considerably narrower shape-invariant beams accelerating (bending) along arbitrary trajectories.

## Results

### Generating non-diffracting beams through gravitational collimation

**Figure 1:**
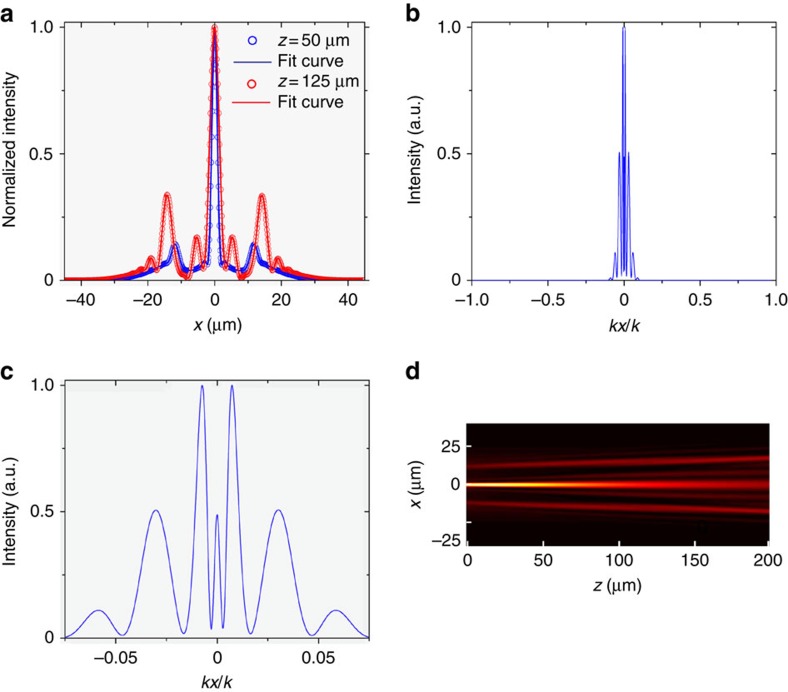
Calculated propagation of gravitational collimation resulting in a non-diffracting beam. (**a**) The calculated non-diffracting beam fitted to the beam arising from the simulation of the experimental setting. (**b**) Spatial spectrum of the beam displaying two main peaks, as can be seen in **c** showing zoom-in on the central section of the spectrum. The two pronounced peaks correspond to a superposition of non-diffracting cosine and sine distributions, resulting in the narrow non-diffracting beam. (**d**) Simulated propagation of the non-diffracting beam of **a**, for a distance of 200 μm inside a homogenous medium, revealing the non-diffracting property.

The first goal is to create a narrow beam that would propagate in a non-diffracting fashion for a considerable distance in a homogeneous medium. We do that by passing a Gaussian beam through a specific refractive index structure, inspired by the gravitational lensing phenomenon occurring around massive stars. We design a specific curvature where the emulated gravitational lensing of the light on the micro-scale can create a very narrow non-diffracting beam. The basic principles of diffraction imply that non-diffracting beams can be constructed when their plane-waves constituents accumulate phase at the same rate. The non-diffracting property of beams depends on the dimensions of the wavepackets, that is, a non-diffracting beam can be a shape-invariant solution to the wave equation in three dimensions (3D) or in two dimensions (2D). In 3D homogeneous media, beams that are structured in both their transverse dimensions exhibit shape-invariant propagation on a straight line in the third dimension include the family of Bessel beams^1^. In 2D, on the other hand, when the beams are structured in a single transverse dimension (for example, when the beam is propagating in a planar waveguide), an ideal non-diffracting beam has a unique shape: two plane waves propagating at opposite symmetric angles with respect to the propagation axis. However, whereas the Bessel beams are localized, that is, they have a main lobe carrying most of the power, the planar case is just an interference grating—which is periodic and cannot be used for applications that require a beam with a single main lobe. Interestingly, providing proper spatial bandwidth to each of the opposite waves in the one-dimensional (1D) case does lead to a localized beam displaying non-diffracting features for some finite distance. More specifically, superimposing two beams whose spectrum in *k*-space is small compared with the wavenumber, at opposite angles with respect to the propagation axis, gives rise to non-diffracting propagation up to a finite distance, due to the similar rate of phase accumulation of the different modal (plane waves) constituents. Here, we construct such a very narrow non-diffracting beam by drawing on intuition from GR, where it is known that light waves are deflected by the space curvature generated by a massive star^63^,^64^. We exploit this gravitational lensing effect to construct a field that is a superposition of two beams of a finite spatial bandwidth, propagating at opposite angles with respect to the propagation axis. Such a beam remains non-broadening for a finite distance that can be much larger than the Rayleigh length of its main lobe. An example for such a 1D non-diffracting beam and its spectrum is displayed in Fig. 1a,b, respectively. Figure 1c shows zoom-in on the spectrum, while Fig. 1d presents its simulated propagation—where it is clear that the main lobe remains narrow for a large distance, in spite of the fact that its width is only four wavelength. The two main peaks in the spectrum (Fig. 1c) represent a superposition of cosine/sine distributions, along with a central peak. The width of the spectral peaks is two orders of magnitude smaller than the wavenumber, enabling a non-diffracting property to a finite distance. This structured beam, whose full-width-half-maximum (FWHM) is 2 μm, is approximately shape-preserving for ∼200 μm, which corresponds to six Rayleigh lengths (Fig. 1d).

**Figure 2:**
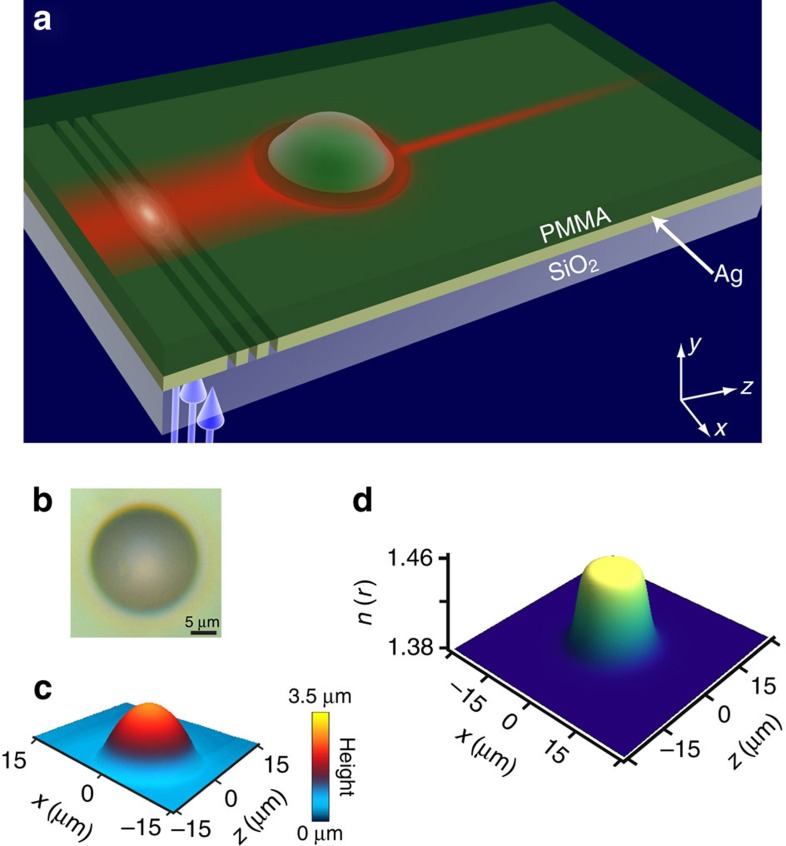
The sample fabricated for generating a narrow collimated beam. (**a**) Schematic view of the fabricated waveguide: the inhomogeneous planar waveguide with the specifically designed refractive index structure. The structure is fabricated by depositing a thin silver film on a silica (SiO_2_) substrate with a thickness of 80 nm, followed by PMMA microsphere powder scattered on the substrate. The blue arrows at the bottom represent the incident 457 nm blue laser light, and the bright spot marks the illumination spot where the light is incident on the grating. (**b**) Top-view optical microscopy image of the microdroplet. (**c**) The surface structure of the microdroplet, as mapped by AFM measurements. (**d**) The effective refractive index structure calculated from **c**, based on waveguide theory.

To transform a broad Gaussian beam (FWHM ∼30 μm) into this non-diffracting beam in a planar waveguide setting, we fabricate a specific refractive index structure inspired by the concepts of curved space known from GR. Namely, curved space generated by a massive gravitational body leads to gravitational lensing, that can in principle overcome diffraction broadening and cause beam collimation. The planar waveguide has a unique width profile, causing a change in the propagation constant and effectively modifying the refractive index. The structure is shown in Fig. 2a. During the fabrication process, a silver film is deposited on a silica (SiO_2_) substrate with a thickness of 80 nm, followed by polymethyl methacrylate (PMMA) microsphere powder scattered on the substrate. The microspheres are distributed on the substrate, with a small density and large separation distance between microspheres. The sample processing includes a stage where the sample is put on the heating table (300 °C) for 30 s. As the melting temperature of PMMA polymer is ∼250 °C, the heating process deforms the PMMA microspheres into domes, just as shown in Fig. 2b,c. In this process, the size of resultant PMMA domes is not uniform, and their diameters can vary greatly, from 1 to 100 μm. For the experiment presented here, we work with one of domes that has an appropriate size, as shown in its optical microscope image in Fig. 2b. The structure is shaped as a dome protruding from the plane of the waveguide (Fig. 2a). This is further confirmed by mapping the surface structure with atomic force microcopy (Asylum Research, MFP-3D-SA, USA), as shown in Fig. 2c. Next, a set of gratings with the period 310 nm are drilled on the sliver film around the microdroplet with focused ion beam (FEI Strata FIB 201, 30 keV, 150 pA). These gratings enable to couple the light into the slab waveguide. Next, we spin-coat the sample with a PMMA photoresist mixed with rare earth (Eu^3+^) to a thickness of ∼1 μm, and subsequently dry the sample in the oven at 70 °C for 2 h. The Eu^3+^ rare earth ions are added to the sample to facilitate fluorescence imaging that will reveal the propagation dynamics of the beam. These Eu^3+^ ions absorb the beam propagating in the slab waveguide, whose wavelength (457 nm) is specifically chosen to excite the rare earth ions, that in turn emit fluorescent light at 615 nm wavelength. We note that, although the 1-μm-thick PMMA layer is not single-mode waveguide for the 457 nm beam, the designed grating allows only one mode to be excited inside the waveguide. Here, only the TM3 mode is excited in our experiment (The grating is designed that only one waveguide mode is excited. Hence, plasmonic modes are not excited in the experiment). The resultant 2D structure of the refractive index is displayed in Fig. 2d, together with a 3D illustration of the entire sample (Fig. 2a). Figure 2c shows the width of the PMMA waveguide as mapped by AFM measurements. From this width, we calculate the refractive index structure displayed in Fig. 2d, which is fitted with the function 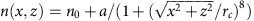, with *n*_0_=1.37, *a*=9.22 × 10^−2^, *r*_*c*_=9.69. Recall that the refractive index of bulk PMMA polymer is 1.49, hence our fabrication process reduces the refractive index according to our design. Specifically, in the region of the dome, the thickness is increased to 3.5 μm, and therefore the effective index of the TM3 waveguide mode is increased from 1.37 to 1.49.

**Figure 3:**
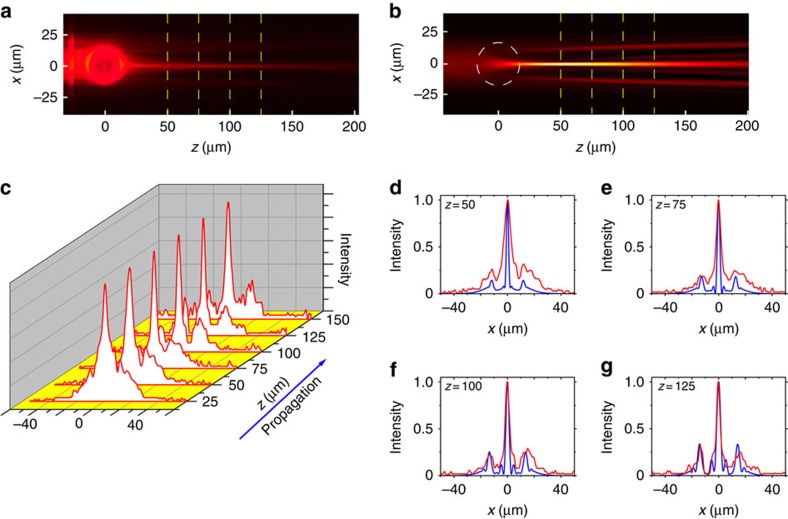
Experimentally observed propagation dynamics of gravitationally collimated non-diffracting beam. (**a**) Top-view photograph of the experimentally observed results obtained through florescence. A broad Gaussian beam with FWWH 11.3 μm passes through the region of the dome, giving rise to the refractive index profile described in Fig. 2c. The wide Gaussian beam focuses to a narrow collimated beam that is non-diffracting for ∼200 μm. The entire beam transformation process occurs within20 μm. (**b**) Simulated results of the same beam showing a similar effect as the experiment. The white dashed circle corresponds to the dome region. (**c**) Normalized intensity profile of the beam for several propagation distances, after passing though the dome region. (**d**–**g**) Measured (red) and simulated (blue) 1D intensity profiles for *z*=50 μm, *z*=75 μm, *z*=100 μm, *z*=125 μm, respectively, which correspond to the planes marked by the yellow dashed lines in **a**–**b**.

In the experiment, we launch a Gaussian beam of 457 nm wavelength and 11.3 μm FWHM to propagate inside the PMMA layer that acts as a waveguide. The loss in this waveguide is quite small, in spite of the proximity of the thin Ag layer, enabling propagation distances of hundreds of micrometres. The specifically designed refractive index structure focuses the wide beam to a very narrow (2 μm) beam that is subsequently propagating without diffraction for ∼200 μm, as expected from the theory. We emphasize that, after passing the ‘star', the very narrow beam is propagating in a completely homogeneous medium, hence its non-diffracting property arises solely from the beam structure generated by passing the ‘star'. Moreover, whereas most shape-preserving beams are very broad, this beam presents a narrow profile, only 2 μm wide. For comparison, we study the dynamic of a Gaussian beam passing through the same medium numerically and compare it with the experimental results (Fig. 3). We do this by numerically simulating the beam propagation, with the beam propagation method in a medium with the specific refractive index structure conforming to that of the sample used in the experiment (Fig. 2d). In both the experiments and the simulations the transformation of the wide Gaussian beam to a narrow collimated beam is achieve within a very short propagation distance (∼20 μm), allowing the use of this scheme in integrated photonics circuits. In Fig. 3, the diameter of the dome is roughly 25 μm. In the experiment, we can fabricate domes with different diameters, always with circular shape. Naturally, domes of different sizes yield collimation for different propagation distances and with different beam widths.

### Experiments emulating the Einstein rings phenomenon

**Figure 4:**
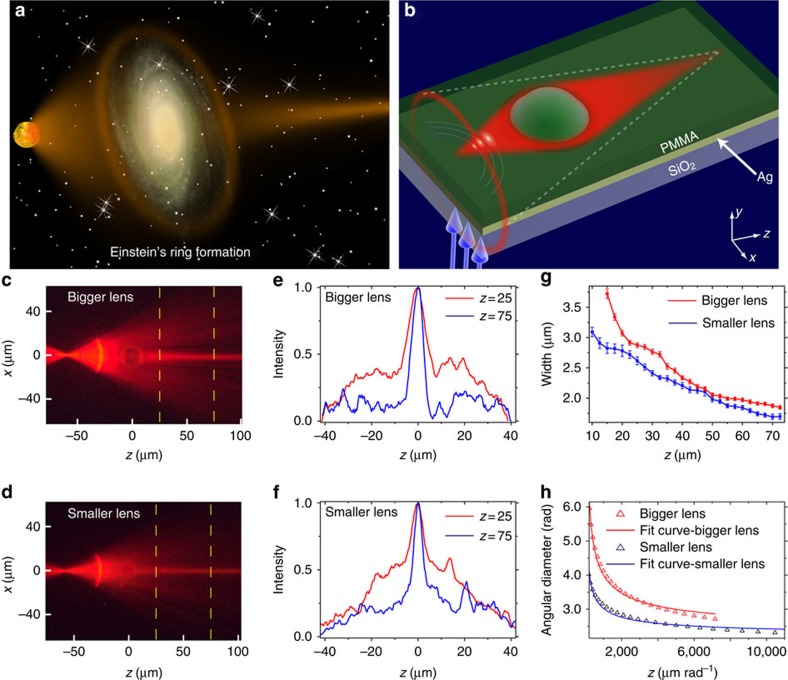
Experimental emulation of the formation of Einstein's ring. (**a**) Einstein's vision: light from a point source is focused by a gravitational lens, and is subsequently observed as a virtual ring around the mass distribution. (**b**) Schematic view of the fabricated inhomogeneous waveguide. (**c**,**d**) Experimental results (obtained through florescence) showing a spherical wave passing though the dome region, for two domes of different radii. The inhomogeneous area acts as a gravitational lens on the light. (**e**,**f**) Measured beam profiles at *z*=25 (red line), *z*=75 (blue line), respectively, which correspond to the locations marked by the yellow dashed lines in **c**,**d**. (**g**) Measured beam width as a function of the propagation distance in the homogenous medium after the dome region. (**h**) Fit to Einstein's formula for the angular diameter of the Einstein rings. The calculated angular diameter from the experimental measurement is in a very good agreement with the theoretical formula.

Interestingly, we find that besides producing collimated beams, the same planar ‘central potential' index structure can also be used to emulate the phenomenon of Einstein's Rings, which is a famous phenomenon predicted by GR and observed in astronomy^65^,^66^. The Einstein Ring phenomena occurs when light from a point source is deformed by a mass distribution through gravitation lensing that causes the appearance of a ring around the mass distribution. For this case, the beam approaching the ‘star' should emulate the radiation originating from a point source, namely, the wave reaching the ‘star' should be a spherical wave. To emulate a point source, we fabricate (with focused ion beam) an arc-shaped grating (period of 310 nm) inside the metal film. This is shown in Fig. 4b, where the radius of the arc is 30 μm. When a plane wave is incident (from below) on the arc grating, the grating transforms it into a spherical wave propagating inside the waveguide layer. The region of incidence on the grating acts as a point source, emitting a spherical wave diverging both to the left and to the right of that point (negative and positive *z*, respectively). In such a setting, the spherical wavefront produced by the arc-grating emulates the wave radiated outwards from a point source located at the centre of grating arc. When this 1D spherical wavefront is passing by the star—it is focused and the beam width changes as a function of the propagation distance, as extracted from the experimental data. It is important to emphasize that our optical setting represents Einstein's rings formed by a time-harmonic EM waves, hence the entire dynamics is in space (not in time). Typical results for two different ‘stars' (microdroplets with two different radii) are displayed in Fig. 4. As the Radius of the ‘star' is larger the convergence of the beam is more extreme, but the final beam is wider (Fig. 4). At this point it is intriguing to compare our emulation results with Einstein's prediction. The Einstein Formula for the angular diameter of the virtual ring^64^ is given by 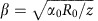, that depends on the convergence angle *α*_0_, the radius of the mass distribution *R*_0_ and the distance between the centre of the mass distribution to the observation point. We calculate the angular diameter of the Einstein Ring from the measured convergence angle of the beam, for several different observation points (propagation distances). For a given observation point, the focusing angle of the beam after passing the ‘star' gives the slope, from which we calculate the angular diameter of the virtual ring that an observer located at this specific distance (from the ‘star') will see. To conform with the Einstein formula, we calculate the relative angular radius between the two mass distributions (two samples). Namely, instead of calculating the absolute angular radius as a function of *z*, we calculate the relative angular radius between the results of each sample. We then fit the curve 
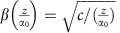
 with *c* as a free parameter and compare the relative constant extracted from the experiment with the constant expected from Einstein's formula. In comparing the ratio and not the absolute number, we avoid the factor 2 between the relativistic Einstein formula and our experiment that represents Newtonian dynamics. As Fig. 4h shows, the experiments agree well with theory, although at large *z*, the experimental values are slightly lower than the model. This minute discrepancy arises from the difference between the fabricated optical potential (refractive index structure) and the 1/*r* gravitational potential of a point source. Consequently, for large values of *z* (distances), the focusing angle of the light deviates from Einstein's formula, hence the measured focusing angle is somewhat smaller than the theoretical curve.

### Shaping beams accelerating on arbitrary trajectories

Finally, we present a general formalism for transforming broad Gaussian beams to accelerating beams that bend along arbitrary (convex) trajectories in a planar waveguide setting. As above, we do that by passing an incident broad Gaussian beam (11.3 μm FWHM) through a miniature refractive index structure that is designed specifically for this task. Accelerating beams are beams with a well-defined peak intensity that propagates along some non-straight trajectory, depending on the phase of the initial beam^4^,^5^,^29^. From the point of view of GR, the peak intensity of the beam does not follow geodesics paths^67^, which are the shortest paths that light propagated along (by the Fermat principle). This important property of accelerating beams had been exploited for various applications, such as curved plasma channels^65^, manipulating microparticles^68^,^69^ and micromachining^70^. We design accelerating beams by utilizing the formalism suggested in ref. 5, for finding the specific 1D phase *φ*(*x*) required for shaping the wavefront of an accelerating beam that will propagate along a specific trajectory. This ID phase can be achieved by a 2D refractive index structure that the beam passes through, and obeys the relation


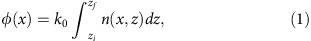


under the assumption that the propagation of the beam is in the paraxial regime. Using this method, there is no unique solution for *n*(*x*, *z*). We therefore suggest a simple method that solves equation (1) for one specific refractive index profile to a specified phase, by assuming *n*(*x*, *z*) is constructed from a function that is separable in *x*, *z*, namely *n*(*x*, *z*)=*f*(*x*)*g*(*z*). For simplicity, we take *g*(*z*)=exp(−*z*^2^/σ^2^), and assume the Gaussian width (in *z*) is small compared with the propagation distance (*σ*<<*z*_*f*_−*z*_*i*_). This allows setting the boundaries of the integral to infinity which after integrating over *z* yields:


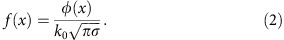


**Figure 5:**
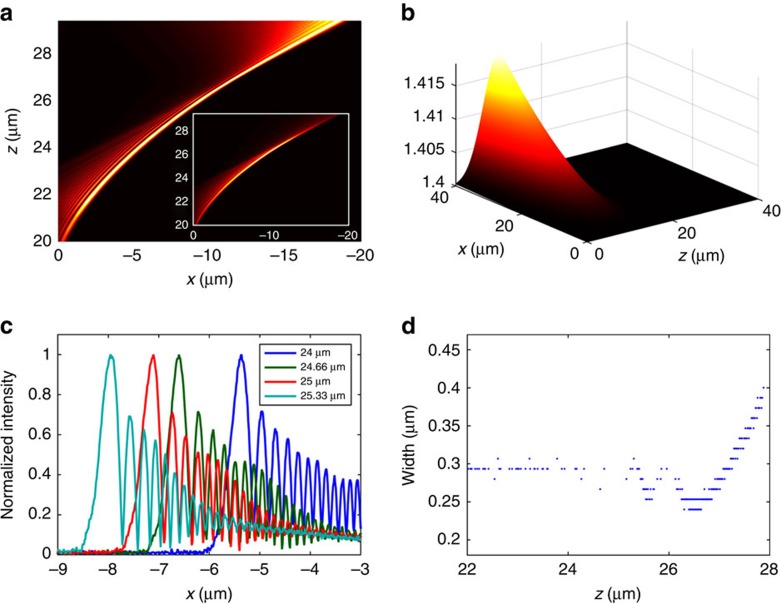
Accelerating beams propagating along arbitrary trajectories produced by designing the refractive index structure within the initial 10 μm propagation distances in the waveguide layer. (**a**) Simulated evolution of the accelerating beam, where the peak intensity is propagating along the curve *f*(*z*)=*az*^3^. Inset: the evolution displayed with a non-normalized intensity (**b**) The calculated refractive index structure which transforms a broad Gaussian beam into the narrow non-diffracting accelerating beam of **a**. (**c**) Structure of the accelerating beam for different propagation distance. (**d**) Width of the main lobe as a function of the propagation distance.

It is important to emphasize that the approximation we used for solving the integral of the phase only, causes additional effects. Due to the 2D refractive index distribution the beam is shifted to some different direction of propagation—*z*'=*ze*^*iθ*^ while propagating through the inhomogeneous area. Consequently, *n*(*x*, *z*)=exp(−*z*^2^/*σ*^2^). To present an example for this method, we find the refractive index profile required to create the phase for an accelerating beam along the trajectory *f*(*z*)=*az*′^3^. In this specific case, the propagation of the resulting beam can be solved analytically using the method presented in ref. 5. In more complicated cases, a numerical solution for the ordinary differential equation (ODE) is required. We then use equation (1) to calculate the 2D refractive index structure that will provide the beam with the appropriate phase. By simulating the dynamic of a broad Gaussian beam passing through the designed refractive index structure, we find that the main lobe indeed accelerates along the expected trajectory, for a distance of 20 μm as displayed in Fig. 5. In this regime, it is possible to design a beam that will accelerate beam on an arbitrary trajectory. As any accelerating beam, the structure of such a beam involves a main lobe accompanied by oscillations on one side, and exponential decay on the other side. An example is shown in Fig. 5c, where the beam cross-sections at several propagation distances is displayed. This technique for beam shaping inside a slab waveguide is general, and can be used to shape the wavefront of non-diffracting beams accelerating on any convex trajectory, by designing the refractive index structure using equations (1 and 2), which relates the initial phase front (assumed here to be of a broad Gaussian beam) and the desired phase front *φ*(*x*) to the refractive index structure required for such wavefront shaping.

## Discussion

To conclude, we have presented a method for shaping optical wavefronts in waveguide settings. Our technique is inspired by GR and it provides a platform for emulating the spatial dynamics of EM waves in curved space. This method can be achieved in thin film waveguides and can be implemented in integrated photonics settings. Specifically, we have demonstrated experimentally the construction of a narrow non-diffracting beam, the formation of Einstein's rings, and presented a general method to construct accelerating beans propagating along arbitrary trajectories. This method can be used for shaping any general beam, thereby suggesting a new way of using transformation optics media for beam shaping in waveguide settings with a single dielectric material. In this work, we presented beam shaping in the spatial domain; consequently, our experiments employed only continuous laser beams as our input waves. However, in principle this technique can also be used to shape ultrashort laser pulses with the traditional grating pairs, the lenses and the spatial modulation at the focal plane, all implemented in a slab waveguide geometry with proper design of the planar refractive index structure. This idea will be pursued in our future research.

## Additional information

**How to cite this article**: Sheng, C. *et al.* Wavefront Shaping through Emulated Curved Space in Waveguide Settings. *Nat. Commun.* 7:10747 doi: 10.1038/ncomms10747 (2016).
